# Characterization of Circulating Immune Complexes in Patients with Myeloperoxidase–Antineutrophil Cytoplasmic Antibody-Associated Glomerulonephritis

**DOI:** 10.3390/ijms27104303

**Published:** 2026-05-12

**Authors:** Takashi Oda, Iroha Okano, Hiyori Takahashi, Azumi Nara, Sachiko Iwama, Takahiro Uchida, Muneharu Yamada, Kazuo Yamakami, Tadasu Kojima

**Affiliations:** 1 Department of Nephrology and Blood Purification, Kidney Disease Center, Tokyo Medical University Hachioji Medical Center, Hachioji, Tokyo 193-0998, Japan; 2 Department of Practical Pharmacy, Tokyo University of Pharmacy and Life Sciences, Hachioji, Tokyo 192-0392, Japan

**Keywords:** anti-neutrophil cytoplasmic antibody (ANCA), myeloperoxidase, circulating immune complexes (CICs), complement, classical pathway

## Abstract

Circulating immune complexes (CICs) are frequently detected in the sera of patients with myeloperoxidase (MPO)–antineutrophil cytoplasmic antibody (ANCA)-associated glomerulonephritis (MPO-AAGN) and their role in activating the classical complement pathway has been suggested. However, the precise composition and functional characteristics of CICs in AAGN remain poorly understood. We analyzed serum samples from four patients with MPO-AAGN and confirmed CICs. An immunoadsorbent column was prepared using a monoclonal rheumatoid factor antibody that binds to CICs, enabling extraction via immunoprecipitation. CICs were analyzed by Western blotting to detect MPO and IgG. Their capacity to activate complement was assessed in vitro by measuring complement activation products using the enzyme-linked immunosorbent assay following incubation with normal human serum. Western blotting revealed distinct bands for both MPO and IgG in all samples. Incubation of extracted CICs with normal human serum resulted in elevated levels of complement activation products, including C5a and C5b-9. This increase was completely inhibited by the addition of EDTA or EGTA. In conclusion, CICs in the serum of patients with MPO-AAGN contain MPO and IgG (presumably MPO-ANCA). These CICs can activate the complement system, likely through the classical pathway. Our findings support the hypothesis that CICs composed of MPO and MPO-ANCA contribute to complement activation via the classical pathway and play a significant role in AAGN pathogenesis.

## 1. Introduction

Antineutrophil cytoplasmic antibody (ANCA)-associated glomerulonephritis (AAGN) is a kidney disease characterized by severe glomerular inflammation and the presence of ANCA, which is commonly associated with rapidly progressive glomerulonephritis. Despite advances in our understanding and treatment of AAGN, clinical outcomes remain suboptimal, with poor renal and overall prognoses. Therefore, elucidating the pathogenesis of AAGN and developing more effective and less toxic therapies remains a critical clinical challenge [1].

Histologically, AAGN typically presents as pauci-immune necrotizing glomerulonephritis, marked by minimal or absent immunoglobulin and complement deposition [2]. Accordingly, the role of the complement system in AAGN was considered negligible. However, recent studies using animal models and analyses of human clinical samples have revealed that complement activation—particularly via the alternative pathway—plays an important role in AAGN pathogenesis [3,4,5,6,7,8], leading to the clinical application of the C5a receptor inhibitor avacopan [9,10].

Most previous studies have suggested that the alternative pathway is the primary mechanism of complement activation involved in the pathogenesis of AAGN [3,5,8]. Recently, however, we reported that circulating immune complexes (CICs) were detected at a high rate (65%) in patients with myeloperoxidase (MPO)-AAGN using an enzyme-linked immunosorbent assay (ELISA)-based monoclonal rheumatoid factor (mRF) assay [11]. These CIC levels strongly correlated with serum C5a and C5b-9 levels, which are terminal components of the complement cascade. Additionally, CIC levels negatively correlated with results from the WIESLAB^®^ Complement System Classical Pathway assay (Svar Life Science Co., Malmö, Sweden), which decreases in response to classical pathway activation. These findings suggest that CICs trigger classical pathway activation, leading to downstream complement activation [11]. Besides our previous report, Juto et al. demonstrated that plasma C4d levels are significantly elevated in ANCA-associated vasculitis (AAV), particularly in patients with active disease, suggesting involvement of the classical/lectin pathway in systemic AAV [12]. However, they did not observe C4d deposition in renal tissue, arguing against a direct role in renal lesions. By contrast, other studies using electron microscopy [13] and immunofluorescence analysis [7] have demonstrated that immune complex deposition is frequently observed in the kidneys of patients with AAGN. These findings strongly support the involvement of the classical complement pathway in renal lesions and suggest that this mechanism may define a subgroup characterized by immune complex-associated pathology. Notably, such deposition has been reported to correlate with clinical parameters, including proteinuria, the extent of crescent formation [13], and renal prognosis [7].

However, the composition of these CICs and whether they truly possess a complement-activating capacity remain unclear. This study aimed to extract CICs, characterize their components using Western blotting, and evaluate their potential to activate the complement system.

## 2. Results

### 2.1. Detection of MPO in CICs from MPO-AAGN Patients

**Figure 1:**
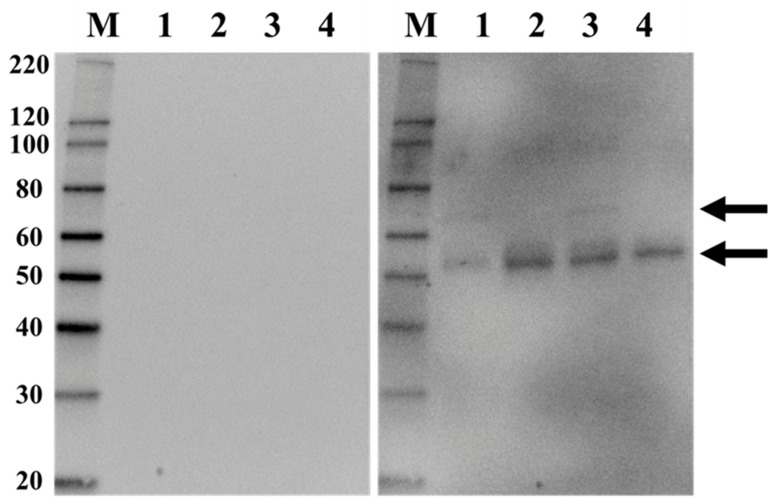
Western blot analysis of isolated circulating immune complexes (CICs) for myeloperoxidase (MPO). CICs were isolated from the serum of four patients by immunoprecipitation using a monoclonal anti-rheumatoid factor antibody and analyzed for MPO by Western blotting. No bands were observed in blots incubated with the secondary antibody alone (HRP-conjugated donkey anti-mouse IgG). Specific bands corresponding to the predicted MPO molecular weight (indicated by arrows) were detected in all four samples when the blots were incubated with the primary antibody. The images on the left and right were captured under identical conditions (single exposure for 5 s). M: MagicMark™ XP Western Protein Standard (Thermo Fisher Scientific, Tokyo, Japan).

Based on our previous findings [11], we hypothesized that CICs in the serum of AAGN patients contain MPO and MPO-ANCA (anti-MPO IgG). To test this, we isolated CICs from the serum of AAGN patients using an immunoadsorbent column and a mouse mRF antibody that binds to the Fc portion of IgG within CICs. Isolated CICs were then analyzed by Western blotting with an anti-MPO antibody, which revealed bands at the expected size of the MPO α subunit (50–60 kDa) in all samples and additional bands corresponding to the MPO α/β intermediate form (60–80 kDa) in samples 1 and 3 (Figure 1, arrows). No bands were detected in the secondary antibody, only controls. These results suggest that the samples isolated using the immunoadsorbent column with mRF antibody contain MPO.

### 2.2. Detection of IgG (Presumed MPO-ANCA) in CICs

**Figure 2:**
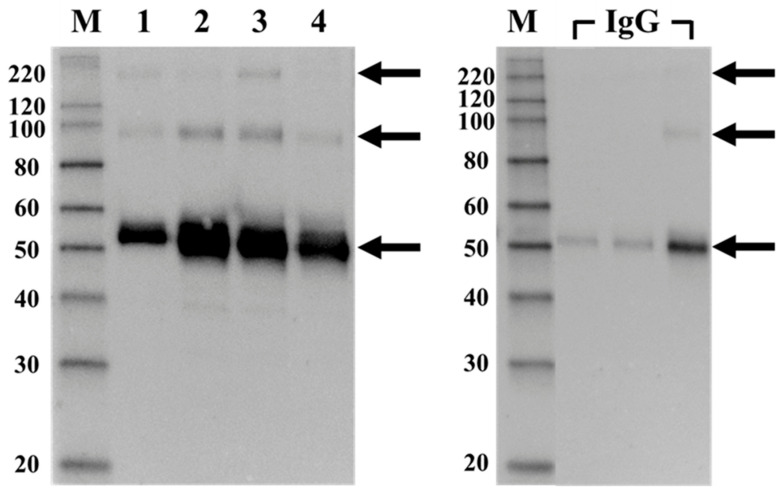
Western blot analysis of isolated circulating immune complexes (CICs) for human IgG. CICs isolated from the serum of four patients were analyzed by Western blotting for human IgG (left panel). Commercially available normal human IgG (FUJIFILM Wako Chemicals, Tokyo, Japan), serially diluted, was electrophoresed as a positive control and analyzed by Western blotting (right panel). Distinct bands corresponding to the expected molecular weight of human IgG (indicated by 3 arrows: 50–60 kDa, 90–100 kDa, and 220 kDa) were detected in all four samples, showing the same pattern as that of the commercial IgG used as the positive control (right panel, 3 arrows of similar size). The images in the left and right panels were captured under identical conditions (single 2 s exposure). M: MagicMark™ XP Western Protein Standard (Thermo Fisher Scientific).

We further analyzed the existence of IgG in the isolated CICs by Western blotting with anti-human IgG, which revealed strong bands at 50–60 kDa in all samples, with additional bands near 90–100 kDa and 220 kDa (Figure 2, arrows). These bands corresponded to those observed in the positive control (commercial human IgG), indicating that they represent IgG isoforms. Thus, the isolated CICs were suggested to contain IgG (presumably at least in part MPO-ANCA).

### 2.3. Complement Activation by CICs

**Figure 3:**
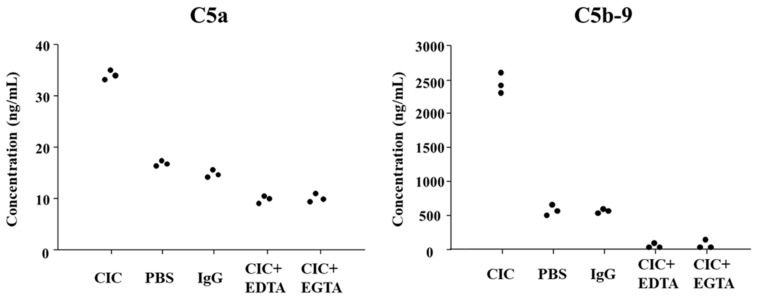
Complement-activating capacity of isolated circulating immune complexes (CICs) in vitro. Incubation of CICs with normal human serum led to increased concentrations of C5a and C5b-9 compared with controls treated with PBS or normal human IgG. These increases were completely abolished by the addition of EDTA or EGTA. The dots show the values of triplicate assays in each group.

To assess the complement-activating capacity of the extracted CICs, we incubated them with normal serum. Incubation of CICs with normal serum led to marked increases in C5a and C5b-9 levels compared to the controls [phosphate-buffered saline (PBS) or IgG alone]. These increases were entirely inhibited by the addition of EDTA or EGTA, suggesting that complement activation occurred via a classical pathway (Figure 3).

## 3. Discussion

MPO-ANCAs can be classified into high-affinity and low-affinity types based on their affinity for the target antigen (MPO). We previously demonstrated that this difference correlates with disease activity and clinical features of AAGN [14,15]. Although AAGN is typically characterized as a pauci-immune disease, we detected CICs at high frequency in MPO-AAGN patients [11]. Notably, in all CIC-positive cases, ANCAs belonged exclusively to the high-affinity type. Specifically, CIC positivity, as assessed by the mRF assay, was significantly associated with elevated MPO-ANCA affinity. Based on these findings, we hypothesized that CICs in AAGN were composed of MPO and MPO-ANCA. In the present study, we confirmed this hypothesis by extracting CICs from sera of patients with MPO-AAGN using immunoprecipitation with the mRF antibody and analyzing their composition by Western blotting.

MPO is a homodimeric protein with an α_2_/β_2_ structure, in which the α subunit weighs 59 kDa and the β subunit weighs 13.5 kDa. The transient intermediate α/β form weighs approximately 74 kDa, while the complete homodimeric α_2_/β_2_ form weighs around 150 kDa [16]. In our Western blot analysis using anti-MPO antibody, a prominent band at 50–60 kDa was observed in all samples, likely corresponding to the α subunit of MPO. Additionally, weaker bands at 60–80 kDa were detected in some samples, potentially representing the transient α/β intermediate form. No bands were observed when only the secondary HRP-labeled donkey anti-mouse IgG antibody was used, confirming that the mouse-derived mRF antibody employed during immunoprecipitation was effectively excluded from the final CIC extract. This result demonstrates the high specificity and reliability of our immunoprecipitation method for isolating CICs from patient sera.

Western blot analysis utilizing anti-human IgG antibody revealed bands at 50–60 kDa, 90–100 kDa, and 220 kDa in commercially sourced normal human IgG, which served as our positive control. Comparable banding patterns were evident across all patient samples, thereby confirming IgG as an integral component of the CICs. Considering the concurrent presence of MPO within these samples, it is reasonable to conclude that a proportion of the detected IgG comprises anti-MPO antibodies (MPO-ANCA).

Furthermore, evaluation of the complement-activating potential of the extracted CICs in vitro showed that incubation with normal serum led to increased levels of the complement activation products C5a and C5b-9 compared to controls treated with PBS or normal human IgG. These elevations were completely abolished by the addition of EDTA (a chelator of both Ca^2+^ and Mg^2+^) or EGTA (a Ca^2+^-specific chelator), indicating that CIC-mediated complement activation does not occur through the alternative pathway but rather occurs via the classical pathway. These in vitro results align with our previous serological findings in patients with MPO-AAGN, where CIC levels positively correlated with serum C5a and C5b-9 concentrations and negatively correlated with the WIESLAB^®^ Classical Pathway assay results.

As noted in our previous report [11], a major limitation of this study is the small number of patients analyzed, as well as the single-race cohort, which may introduce population bias. There are two main reasons for the limited number of cases analyzed in this study. First, this study was designed to validate our previous study [11], which included 20 patients with MPO-AAGN. From this cohort, we selected only those cases with elevated CIC levels, as determined by mRF-ELISA, and further restricted the analysis to cases for which sufficient serum samples were available. Second, the analysis required preparation of an immunoadsorbent column with immobilized mRF antibodies to isolate CICs from patient serum. However, due to the limited availability of mRF antibodies, it was not feasible to perform extractions on a larger number of cases. Given the limited number of cases analyzed, the generalizability of these findings is limited. Larger-scale studies will be required to clarify the prevalence of this subgroup within the broader AAGN population and to further define its clinical and pathological characteristics.

In conclusion, this study demonstrated that CICs isolated from the serum of patients with MPO-AAGN contain MPO and IgG (presumably MPO-ANCA); notably, these complexes can activate the complement via the classical pathway in vitro. In conjunction with our earlier findings in vivo—namely, the frequent presence of CICs in AAGN patients with high-affinity MPO-ANCA, and the significant correlations between CIC levels and serum C5a, C5b-9, and WIESLAB^®^ Classical Pathway Assay [11]—MPO and MPO-ANCA may form immune complexes that trigger classical pathway activation, contributing significantly to MPO-AAGN pathogenesis. Considering recent advancements in complement-targeted therapies for renal diseases, such as eculizumab [17], avacopan [9,10], and iptacopan [18], these findings may open new avenues for therapeutic strategies involving targeted complement inhibition at the classical pathway in the near future.

## 4. Materials and Methods

### 4.1. Patients and Ethics Approval

Serum samples from four patients with MPO-AAGN were included. These patients had high CIC levels, as measured using the mRF ELISA kit (Nissui Pharmaceutical Co., Ltd., Tokyo, Japan) via SRL, Inc., (Tokyo, Japan) as part of our previous study [11]. All patients were MPO-ANCA positive and had clinical (glomerular hematuria and proteinuria > 0.5 g/gCr) and histological evidence (crescentic glomerulonephritis) of renal involvement. Patients with other causes of nephritis (e.g., IgA nephropathy, lupus nephritis, or drug-induced vasculitis) were excluded.

This study was conducted in accordance with the Declaration of Helsinki and approved by the Tokyo Medical University Research Ethics Committee (T-2020-0302). Written informed consent was obtained from all participants for the use of their clinical data and residual biological samples.

### 4.2. CIC Extraction by Immunoprecipitation

An immunoadsorbent column was prepared using the Pierce™ Protein G IgG Plus Orientation Kit (Thermo Fisher Scientific, Tokyo, Japan). The mouse mRF antibody (provided by the research laboratory of Nissui Pharmaceutical Co., Ltd.) was conjugated to a protein G agarose resin, cross-linked, and blocked. Patient serum was applied to the column to allow for antigen–antibody interactions. The eluent’s absorbance was monitored (NanoDrop™2000c, Thermo Fisher Scientific), and the column was washed repeatedly with 0.1 M phosphate-buffered saline (PBS) until the protein was no longer detectable. This cycle was repeated three times. The bound CICs were eluted and concentrated using a 3 K ultrafiltration spin column (APRO Science Group Co., Ltd., Tokushima, Japan).

### 4.3. Western Blot Analysis of Extracted CICs

CICs isolated from four patients with MPO-AAGN were denatured with 2× Laemmli Sample Buffer (Bio-Rad, Hercules, CA, USA) and separated by sodium dodecyl sulfate–polyacrylamide gel electrophoresis (10% acrylamide). Molecular weight markers (MagicMark™ XP Western Protein Standard, Thermo Fisher Scientific) were run alongside the samples. Proteins were transferred to polyvinylidene difluoride membranes and probed with mouse anti-human MPO (R&D Systems, Tokyo, Japan; clone # 392105) and rabbit anti-human IgG (Dako/Agilent Technologies, Santa Clara, CA, USA). Horseradish peroxidase-conjugated donkey anti-mouse and anti-rabbit IgG secondary antibodies (Thermo Fisher Scientific) were used. Bands were detected by chemiluminescence (Ez West Lumi Plus, ATTO, Tokyo, Japan) using a luminescence imager (WSE-6300H LuminoGraph III, ATTO). For Western blotting to detect MPO, a blot incubated with only the secondary antibody was used as a negative control. In contrast, for Western blotting to detect human IgG, commercially available normal human IgG (FUJIFILM Wako Chemicals, Tokyo, Japan) was electrophoresed as a positive control, and both primary and secondary antibodies were used for detection as per the standard protocol.

### 4.4. Evaluation of Complement Activation by CICs

After incubating isolated CICs with normal human serum, complement activation products of C5a and C5b-9 were quantified through ELISA (QuidelOrtho Corporation, San Diego, CA, USA). Specifically, 2 μL of CIC solution was combined with 100 μL of normal serum diluted fivefold. Control conditions substituted PBS or normal human IgG (FUJIFILM Wako Chemicals) for CICs. Pathway specificity was investigated through the addition of 0.01 M EGTA or EDTA. Following a 1.5 h incubation at 37 °C, complement activation products were quantified according to the manufacturer’s protocol.

## Data Availability

The original contributions presented in this study are included in the article. Further inquiries can be directed to the corresponding author.

## Abbreviations

The following abbreviations are used in this manuscript:

CIC	circulating immune complex
MPO	myeloperoxidase
ANCA	antineutrophil cytoplasmic antibody
AAGN	ANCA-associated glomerulonephritis
AAV	ANCA-associated vasculitis
ELISA	enzyme-linked immunosorbent assay
mRF	monoclonal rheumatoid factor
PBS	phosphate-buffered saline
